# Retraction: Small Protein-Mediated Quorum Sensing in a Gram-Negative Bacterium

**DOI:** 10.1371/annotation/880a72e1-9cf3-45a9-bf1c-c74ccb73fd35

**Published:** 2013-09-09

**Authors:** Sang-Wook Han, Malinee Sriariyanun, Sang-Won Lee, Manoj Sharma, Ofir Bahar, Zachary Bower, Pamela C. Ronald

The authors retract this publication due to concerns about the integrity of strain PXO99Dax21 employed in the study. 

During additional experiments carried out at our laboratory, we discovered that the strain PXO99Dax21 employed in the study was mixed up with another strain in our collection. This compromises the validity of Figures 1, 2 and 3 in the article as we are unsure as to whether the correct strain was employed in the studies. 

We have taken steps to repeat the experiments but we have been unable to validate the knockout mutants generated so far. We have also attempted to replicate the work using strains obtained from another group, but in our hands, those strains did not infect rice plants. 

